# A Simple Subjective Evaluation of Enface OCT Reflectance Images Distinguishes Glaucoma From Healthy Eyes

**DOI:** 10.1167/tvst.10.6.31

**Published:** 2021-05-25

**Authors:** Riccardo Cheloni, Simon D. Dewsbery, Jonathan Denniss

**Affiliations:** 1 School of Optometry and Vision Science, University of Bradford, UK; 2 Ophthalmology Department, Leeds Teaching Hospitals NHS Trust, Leeds, UK

**Keywords:** glaucoma, optical coherence tomography, enface imaging, retinal nerve fiber layer, retinal nerve fiber bundles

## Abstract

**Purpose:**

We present a subjective approach to detecting glaucomatous defects in enface images and assess its diagnostic performance. We also test the hypothesis that if reflectivity changes precede thickness changes in glaucoma there should be reduced correlation between the modalities in glaucoma compared to controls.

**Methods:**

Twenty glaucoma participants and 20 age-matched controls underwent high-resolution OCT scans of one eye. 4 µm-thick enface slabs were constructed through the retina. Enface indices were depths of *first gap* in visible retinal nerve fiber bundles (RNFBs) and *last visible bundle,* subjectively evaluated in six sectors of a 3.5 mm circle around the optic disc. Retinal nerve fiber layer thickness (RNFLT) along the same circle was extracted at angles corresponding to enface indices. Between-group differences were tested by linear mixed models. Diagnostic performance was measured by partial receiver operating characteristic area (pAUC).

**Results:**

*First gap* and *last visible bundle* were closer to the inner limiting membrane in glaucoma eyes (both *P* < 0.0001). Enface indices showed excellent diagnostic performance (pAUCs 0.63–1.00), similar to RNFLT (pAUCs 0.63–0.95). Correlation between enface and RNFLT parameters was strong in healthy (*r* = 0.81–0.92) and glaucoma eyes (*r* = 0.73–0.80).

**Conclusions:**

This simple subjective method reliably identifies glaucomatous defects in enface images with diagnostic performance at least as good as existing thickness indices. Thickness and reflectivity were similarly related in healthy and glaucoma eyes, providing no strong evidence of reflectivity loss preceding thinning. Objective analyses may realize further potential of enface OCT images in glaucoma.

**Translational Relevance:**

Novel enface OCT indices may aid glaucoma diagnosis.

## Introduction

Early diagnosis of glaucoma is desirable to minimize visual impairment,^1^^,^^2^ but the burden of lifelong treatment demands accurate diagnosis with minimization of false positives.^3^^,^^4^ Optical coherence tomography (OCT) has become a mainstay of glaucoma assessment,^5^^,^^6^ however, diagnosis from single examinations remains challenging.^7^^–^^9^ Among several factors, subtle early changes to ocular structures with a diverse anatomy among healthy eyes^10^^,^^11^ and suboptimal usage of collected information^12^^,^^13^ contribute to the imperfect diagnostic capability of OCT.

Conventionally, OCT is employed in glaucoma clinics to evaluate retinal nerve fiber layer (RNFL) thickness. An additional source of structural information is retinal nerve fiber bundle (RNFB) reflectivity, which varies according to integrity and density.^14^^,^^15^ Hyper-reflectivity of RNFBs is due to their highly ordered structure, and loss of reflectivity occurs when RNFB axon cytoskeleton is disrupted.^16^^,^^17^ Changes in RNFL reflectance have been assessed in glaucoma clinics long before the introduction of OCT, either by ophthalmoscopy or fundus photography.^18^^,^^19^ Evidence from animal models suggests that loss of reflectivity may precede measurable reduction of RNFL thickness,^20^^,^^21^ but this has not been consistently replicated in humans.^14^ Recent developments in OCT technology allow us to generate enface images, enabling visualization and quantification of RNFB reflectance. Following dense volumetric scans of the area of interest, transverse enface slabs can be obtained by averaging the intensity of each A-scan over a certain depth below the inner limiting membrane (ILM), producing a two-dimensional image.^22^^–^^26^ As such, enface images allow direct observation of RNFBs, that in healthy eyes appear hyper-reflective due to the ordered structure of ganglion cell axon cytoskeletons.^16^ According to the above models, clinicians could exploit changes in RNFB reflectance as an additional marker of glaucomatous damage.^23^

Further limitations on diagnostic use of OCT for early glaucoma may arise from data analysis. It has been suggested that the current focus on RNFL thickness indices and red/green classification may limit diagnosis as it does not make full use of available information.^13^^,^^27^^,^^28^ Accordingly, clinicians are recommended to look in greater detail at B-scans for evidence of glaucoma damage missed by RNFL thickness analysis.^12^^,^^13^^,^^23^ Enface images may be one way to observe glaucomatous lesions missed by the conventional RNFL thickness approach.

Though the analysis of enface images is promising, the lack of established methodology currently limits clinical value. No accepted objective criteria to define defects in this domain are available and proposed subjective analyses^23^^,^^24^ have not been validated, nor have their diagnostic performance been evaluated.

**Figure 1.**
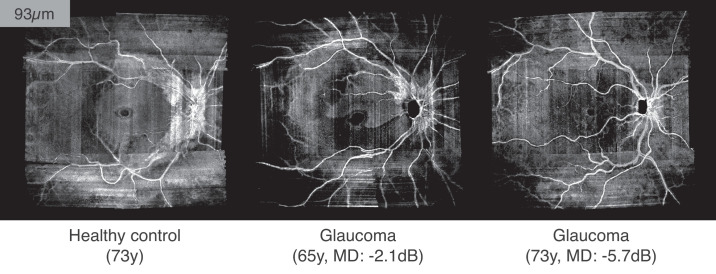
Example of how visible presence of RNFBs (93 µm below ILM) changes in a healthy eye and at different stages of glaucoma. The images are single pixel deep enface OCT images without depth-averaging. At this depth, RNFBs are still visible all around the optic disc in the healthy eye, whereas RNFBs have already disappeared in the rest of the retina where the slab encompasses deeper and hyporeflective retinal layers. In the early glaucoma eye (central panel) a substantial loss of RNFBs can be seen in the temporal and temporal inferior sectors, with no visible presence of RNFBs. In the more advanced glaucoma eye (right panel) no bundles are visible around the optic disc or elsewhere, with the only hyperreflective elements provided by blood vessels. An animated version of this figure, showing a range of depths below the ILM, is provided in Supplementary Figure S1. MD = mean deviation.

The purpose of this study was to evaluate a simple approach for subjective identification of RNFB reflectance loss in glaucoma (Fig. 1 and Supplementary Fig. S1), and to quantify the diagnostic performance of this approach. Further, since the hypothesis that reflectance loss occurs before thickness changes has been minimally investigated in humans, we additionally aimed to test for discordance between RNFB reflectivity and RNFL thickness changes that may indicate a temporal decoupling between these parameters that could be exploited for glaucoma diagnosis.

## Methods

The study adhered to the tenets of the Declaration of Helsinki and received ethical approval from the National Health Service's Research Ethics Service. All participants gave written informed consent and were free to withdraw at any time.

### Participants

Participants were recruited through advertisements placed in the university eye clinic, local eye hospitals, local newspapers and through charities and local interest groups. Recruited participants underwent a detailed eye examination including refraction, Goldmann applanation tonometry, slit lamp examination, spectral domain OCT (Spectralis, Heidelberg Engineering, Heidelberg, Germany) and visual field testing (24-2 SITA-Standard, Humphrey Field Analyzer 3, Carl Zeiss Meditec Inc., Dublin, California). Glaucoma participants were included if older than 40 years and with a confirmed clinical diagnosis of open-angle glaucoma with evidence of both structural and functional (visual field) defects. Structural defects were defined as at least one abnormal sector (*P* < 1%) from the 3.5 mm diameter circumpapillary RNFL (cpRNFL) thickness OCT scan. Visual field defects were defined as at least three contiguous non-edge points with *P* < 5% on the Pattern Deviation plot. Glaucoma participants had no other disease except glaucoma that could affect vision. Age-similar healthy controls were included if presenting with no eye conditions, including ocular hypertension or different intraocular pressure between eyes (>4 mmHg). Healthy participants required normal visual fields (Mean Deviation *P* > 5%, Glaucoma Hemifield Test within normal limits and no visual field defect as defined for the glaucoma group), but no specific OCT criteria were applied. All participants had best corrected visual acuity ≤0.20 logMAR (6/9.5 Snellen), refractive error between ±6.00DS and less than 3.00DC and clear optical media with or without history of uncomplicated cataract surgery in the included eye.

One eye per participant was included. If both eyes were eligible, the included eye was selected at random in controls, whereas the eye with milder defect (as identified by a less negative Mean Deviation) was included among glaucoma participants.

### OCT Imaging & Processing of En Face Images

Details of the OCT imaging procedure have been described previously.^26^ This consisted of seven high-density, high-speed OCT scans (9.65 B-scans per degree), collected in different retinal locations. Overall, the central ±25° of the retina was covered and all images were acquired with signal-to-noise ratio above 20 dB as per the manufacturer's instructions. Single slab images of the maximum digital axial resolution (3.87 µm), containing depth-resolved attenuation coefficients calculated according to equation 17 from Vermeer et al.,^29^ were extracted from 0 to 193.5 µm below the ILM using custom software written in *R* (version 3.6.3).^30^ Attenuation coefficients represent an intrinsic optical property of the retinal tissue^31^ and their use has been proposed to reduce the impact of artifacts on enface images.^22^ Conventional cpRNFL thickness was also measured at the 3.5 mm diameter circle around the optic nerve head (ONH) as segmented by the device's built-in software.

Attenuation coefficient images were imported into MATLAB (Version 9.6.0, MathWorks Inc., Natick, Massachusetts) for montaging and further processing. Custom software was used to produce a montage of single images using the macular scan as a reference image and choosing the highest intensity pixel in regions of overlap between scans. For each participant, obtained montages underwent further image processing to optimize visualization, the details of which have been published.^26^ Briefly, the intensity of an area within the raphe region, 35 µm below the ILM, with no RNFBs was set as background by subtracting this lower limit from all pixels and clipping negative values to zero. Then, the average 99^th^ percentile from all depths was used to normalize the attenuation coefficients arrays to a 0 to 1 range. Figure 1 shows examples of final montages.

### Data Extraction

**Figure 2.**
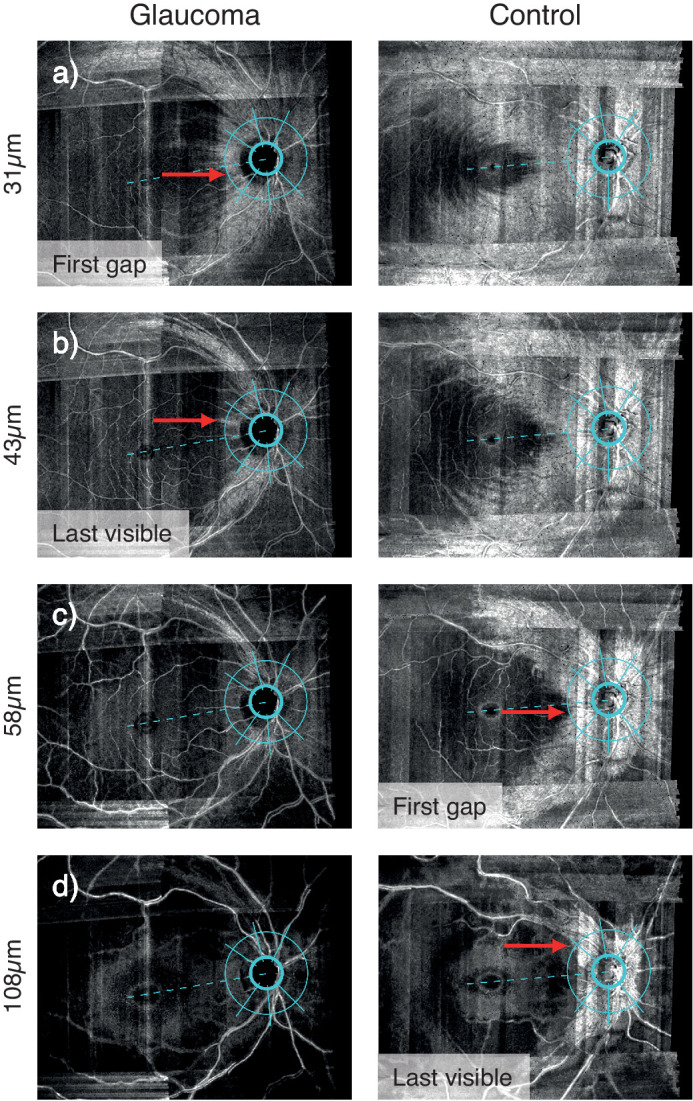
Example of the task in the temporal sector for a glaucoma eye (left panels) and an age-similar healthy participant (right panels). In (**a**) the red arrow shows the first gap for the glaucoma eye at 31 µm below the ILM, whereas the corresponding depth for the healthy eye is reached at 58 µm below the ILM (red arrow in **c**). The depth of last visible bundle (Last visible) was 43 µm (red arrow in **b**) and 108 µm (red arrow in **d**) below the ILM for the glaucoma and healthy eye, respectively.

Images of individual eyes were arranged in presentation files allowing observation of sequential slabs with perfect spatial alignment. Visible presence of RNFBs was evaluated around the ONH in six sectors corresponding to those of the Spectralis 3.5 mm diameter circle scan. The sectors adopted^32^ were the temporal (90°), nasal (110°), two superior and two inferior sectors, split into temporal and nasal (40° per each of four sectors). A sector grid (Fig. 2) with fixed and standardized dimension was overlaid on the enface images of all participants at each depth. Aiming to adopt the same 3.5 mm circle of the cpRNFL thickness analysis, the corresponding scanning laser ophthalmoscope (SLO) image of Spectralis analysis from one participant was used to drive the construction of the grid by overlapping corresponding retinal structures. The resulting grid was subsequently verified on a second participant. Grid dimensions were then preserved unchanged and applied to all assessed eyes except that the grid was tilted to follow the individual fovea-disc angle as subjectively identified in the enface image. Accordingly, the temporal sector of each individual eye was centered on the fovea-disc axis, mimicking the arrangement used in the Spectralis cpRNFL analysis, facilitating comparison (Fig. 2).

Visible presence of RNFBs was recorded subjectively by one of the authors (RC), viewing images on a MacBook Pro 13” computer (2017 version, Apple Inc., Cupertino, California) under standardized lighting. Both the depth and corresponding angle (with 0° at the fovea-disc axis and angles increasing clockwise for right eyes and anticlockwise for left eyes) of two enface indices were extracted at each ONH sector (Fig. 2). **First gap in visible bundles** (Figs. 2a, c), subsequently referred to as *first gap*, refers to the first (most anterior) depth at which a gap between RNFBs can be seen crossing the 3.5 mm circle in the sector of interest. **Last visible bundle** (Figs. 2b, d) represents the most posterior depth at which one or more visible bundles crosses the 3.5 mm circle in the sector of interest.

To reduce measurement bias, the grader was blinded to the depth of each image during the grading task. It was not possible to mask the grader to the disease status since typical glaucomatous arcuate defects originating from the ONH were readily observable while viewing the enface images. However, to minimize effects of preconception, the grading task was performed first in eyes with glaucoma, and subsequently in healthy controls. Hyper-reflectivity from blood vessels was ignored in performing the judgment.

**Figure 3.**
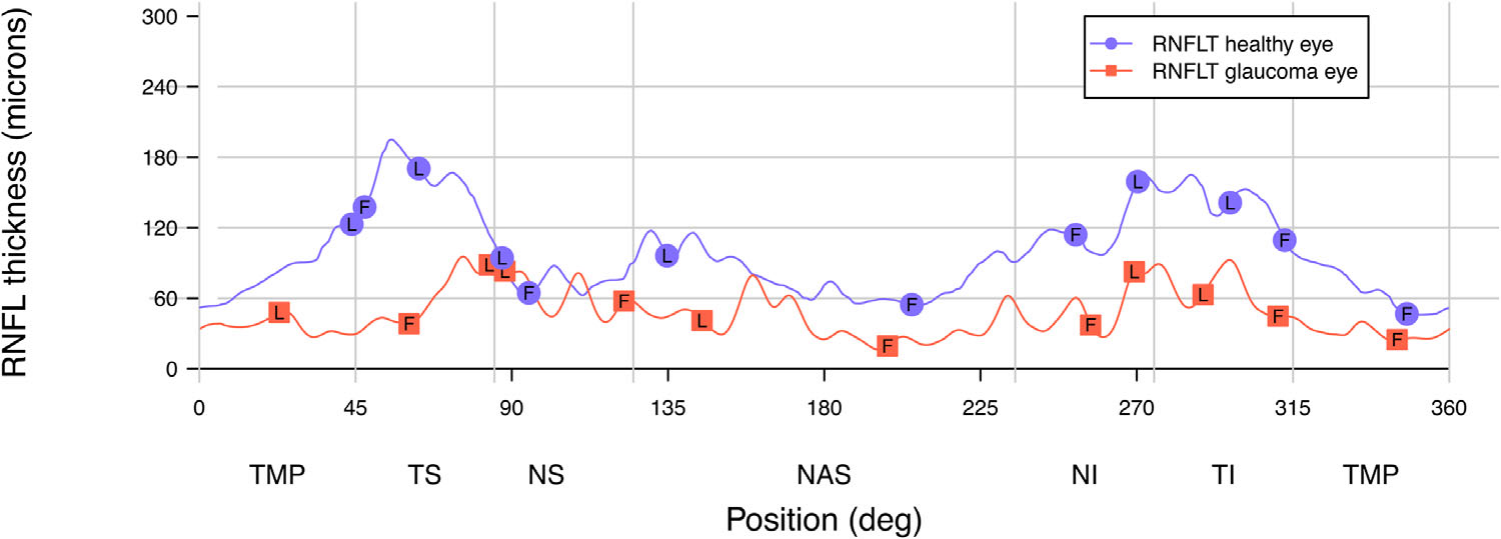
Circumpapillary RNFL thickness (RNFLT) profiles for the same control and glaucoma eye as shown in Figure 2. Points marked by F and L represent the RNFL thickness at the angles corresponding to the enface measures of first gap and last visible bundle, respectively. For both eyes and in every ONH sector, RNFL thicknesses at angle of first gap (F) were smaller than thicknesses at angle of last visible bundle (L), hence appearing lower on the y-axis. TMP = temporal, TS = temporal superior, NS = nasal superior, NAS = nasal, NI = nasal inferior, TI = temporal inferior.

In addition to the enface indices, RNFL thickness along the same 3.5 mm circle was extracted at the angles of the enface parameters in each ONH sector (Fig. 3). Conventional cpRNFL parameters including mean sector thickness and global thickness were also extracted. These data served to establish a comparison between the enface parameters (first gap and last visible bundle) and the conventional thickness measures in our sample. Lastly, the average (mean) first gap and last visible bundle were computed for each eye, weighting for the width of each ONH sector by multiplying the indices by the width of the corresponding sector, summing and then dividing by 360°. Weighted average RNFL thickness at first gap and last visible bundle angles were computed the same way.

### Statistical Analysis

All analyses were conducted in the open-source environment *R* (version 3.6.3)*.*^30^ Linear mixed models^33^ and likelihood ratio tests were used to evaluate the overall effect of glaucoma on first gap and last visible bundle, while accounting for repeated-measures from six ONH sectors within each eye. We tested whether the depth of these enface indices was affected by glaucoma (fixed effect), accounting for the individual eye and the ONH sector as random effects. This analysis was limited to data from individual sectors. Models took the form:

(1) $$\;\;\;\begin{array}{c} y∼1+Disease\;Status+\left(1|eye\right)+\left(1|ONH\;sector\right)+\varepsilon \end{array}$$

where y signifies the measure of interest (first gap or last visible bundle), 1 signifies the intercept and *ε* signifies random error. A model of the same form was applied to counterpart RNFL thickness data. Post-hoc independent *t*-tests were used to evaluate between-group differences in individual sectors, adjusting for multiple comparisons by Bonferroni correction. Diagnostic capability was quantified with standardized partial receiver operating characteristic area (pAUC).^34^ To focus on the highest levels of specificity,^3^^,^^4^ pAUCs with bootstrap 95% confidence intervals (CI) were calculated at specificity between 90% and 100%, with the trapezoid method.^35^ Comparisons between pAUCs of enface indices and corresponding RNFL thickness parameters were made with the DeLong method.^36^ An overall measure of the strength of correlation between depth of visible presence of RNFBs and RNFL thickness was estimated by repeated measures correlation, using the *RMcorr* *R* package,^37^ according to Bland & Altman.^38^^,^^39^ This method allowed us to account for non-independence of data, and provides a measure of strength of common association among individuals (*r*), interpretable as a Pearson correlation coefficient. For consistency, Pearson correlation was also used to assess the strength of individual enface-thickness relationships within each ONH sector. Correlation analyses were further explored according to disease status, testing the hypothesis that if reflectivity loss precedes thinning, correlation would be poorer in glaucoma compared to healthy eyes.

A power calculation suggested that two groups as small as *n* = 5 per group would provide 90% power at α = 0.05 to detect between-group differences of the magnitude found in recent data on global cpRNFL thickness in healthy and glaucoma eyes.^40^ Data from the overall group (*n* = 40) would provide 90% power (α = 0.05) for identification of correlation of at least *r* = 0.48.^41^ On the other hand, when grouping for disease status (*n* = 20), a correlation of at least *r* = 0.65 could be identified, at the same power and alpha.^41^

## Results

We included 20 glaucoma participants and 20 age-similar healthy controls, whose demographics are given in Table 1. Seven recruited controls and 12 recruited glaucoma participants were excluded for not meeting inclusion criteria (e.g., pathology, VF defect; four controls, eight glaucoma), for ptosis affecting imaging and perimetry (two controls), unable to obtain reliable VF (one control, three glaucoma) or unwillingness to undergo extended OCT scans (one glaucoma). On average, glaucoma participants had an early-moderate visual field defect, with the majority (17/20) showing a Mean Deviation (MD) better than or equal to −6 dB. The remaining three participants presented MD of −6.13 dB, −8.62 dB, and −14.9 dB, respectively.

**Table 1.** Demographics of Included Participants. Continuous Data are Summarized as Mean and (Standard Deviation). *P*-Values Are Calculated Using *t*-Tests for Continuous Data or Proportion Tests for Proportions

	Control	Glaucoma	P
N	20	20	–
Male/female	8/12	9/11	1
Ethnicity, caucasian/others	19/1	20/0	1
Age (years)	68.6 (5.0)	69.3 (5.1)	0.66
Mean spherical equivalent (D)	+0.7 (2.0)	+0.0 (1.4)	0.21
SAP Mean Deviation (dB)	0.6 (1.1)	–4.5 (3.1)	<0.0001
Average cpRNFL thickness (µm)	95.1 (9.3)	66.3 (9.4)	<0.0001

**Figure 4.**
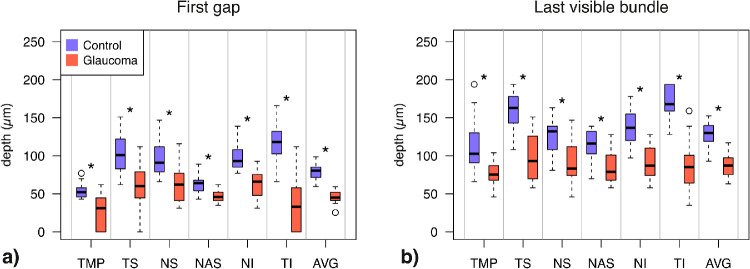
Boxplots showing differences between glaucoma and control eyes for the first gap (**a**) and last visible bundle (**b**) for every ONH sector and the sectors-average. At each ONH sector, control and glaucoma data are reported by the left-most and right-most box, respectively, and color-coded accordingly. After Bonferroni correction (14 comparisons), pairwise differences were considered significant when *P* < 0.0036, and flagged with (*). Boxes report medians and 25^th^ to 75^th^ percentiles. Whiskers represent maximum and minimum values of data within 1.5× interquartile range above or below the limits of the box. Unfilled symbols represent outliers. ONH sectors acronyms as per Figure 3; AVG = average.

Overall, disease status had a significant effect on both enface RNFB indices (χ^2^_(1)_ = 63.3 & 51.6, both *P* < 0.0001). Both first gap and last visible bundle were closer to the ILM in glaucomatous eyes (mean difference: 39.1 µm, 95% CI: 33.0 to 45.3 and 48.1 µm, 95% CI: 38.8 to 57.4 µm, respectively, both *P* < 0.0001). Pairwise differences (Fig. 4) showed both enface indices to be smaller in eyes with glaucoma compared to healthy controls across all ONH sectors (all *P* < 0.0036). The greatest separation was found in the temporal inferior sector for both first gap (difference: 80.8 µm, 95% CI: 62.3 to 98.7 µm; t_35.2_ = 9.1, *P* < 0.0001) and last visible bundle (82.9 µm, 95% CI: 66.0 to 99.9 µm; t_34.2_ = 9.9, *P* < 0.0001). As expected from existing knowledge, linear mixed models showed that RNFL thickness at angles corresponding to enface first gaps and last visible bundles were also significantly smaller in glaucoma (χ^2^_(1)_ = 59.6 & 37.4, both *P* < 0.0001). Pairwise differences among RNFL thickness parameters are shown in Supplementary Figure S2.

Diagnostic performance of enface RNFB indices and RNFL thickness parameters at corresponding angles is reported in Table 2. The performance of conventional cpRNFL thickness analysis in this sample is also reported for comparison.

**Table 2.** Diagnostic Performance (Standardized pAUC at Specificity 90–100% with 95% CIs) of Enface Indices, RNFL Thickness (RNFLT) at Corresponding Angles and Conventional cpRNFL Thickness Measurements. ONH Sector Labels as per Figure 3.

ONH Sector	Enface First Gap	RNFLT at First Gap Angle	Enface Last Visible Bundle	RNFLT at Last Visible Angle	cpRNFL Thickness
TMP	0.86 (0.75, 0.95)	0.82 (0.71, 0.95)	0.63 (0.53, 0.87)	0.67 (0.53, 0.92)	0.70 (0.59, 0.95)
TS	0.79 (0.67, 0.92)	0.79 (0.68, 0.92)	0.80 (0.68, 0.95)	0.76 (0.66, 0.90)	0.74 (0.63, 0.92)
NS	0.82 (0.71, 0.92)	0.67 (0.58, 0.82)	0.76 (0.65, 0.90)	0.65 (0.53, 0.79)	0.72 (0.61, 0.84)
NAS	0.67 (0.55, 0.91)	0.63 (0.53, 0.79)	0.71 (0.57, 0.90)	0.70 (0.59, 0.83)	0.70 (0.55, 0.87)
NI	0.91 (0.83, 0.99)	0.84 (0.71, 0.97)	0.83 (0.71, 0.95)	0.76 (0.66, 0.92)	0.92 (0.82, 1)
TI	0.94 (0.84, 1)	0.88 (0.76, 1)	0.95 (0.87, 1)	0.92 (0.84, 1)	0.95 (0.87, 1)
AVG	1 (1, 1)	0.95 (0.87, 1)	0.90 (0.74, 1)	0.83 (0.72, 0.97)	0.95 (0.87, 1)

Several enface indices showed excellent diagnostic capability (pAUCs > 0.9). The enface first gap indices with best diagnostic performance (inferior temporal and sectors-average) performed slightly better than RNFL thickness counterparts, but they were statistically similar (*P* = 0.18 and *P* = 0.16). Similarly, best performing enface last visible bundle indices (inferior temporal and sectors-average) outperformed corresponding RNFL thickness parameters, but differences were not statistically significant (*P* = 0.33 and *P* = 0.30).

Diagnostic accuracy analysis was repeated in a subgroup of early glaucoma participants with MD better than or equal to −4.0 dB (*n* = 11, Supplementary Table S3). Limiting the analysis to patients with glaucoma at earlier stages aimed to remove more advanced cases that are easier to diagnose.^42^ Nonetheless, pAUCs in the earlier glaucoma group were similar to the ones identified in the overall sample, suggesting no loss of diagnostic performance. Indeed, the best enface and RNFL thickness parameters (temporal inferior and sectors-average) were similar between the overall group and the early glaucoma subgroup (all *P* > 0.05).

**Figure 5.**
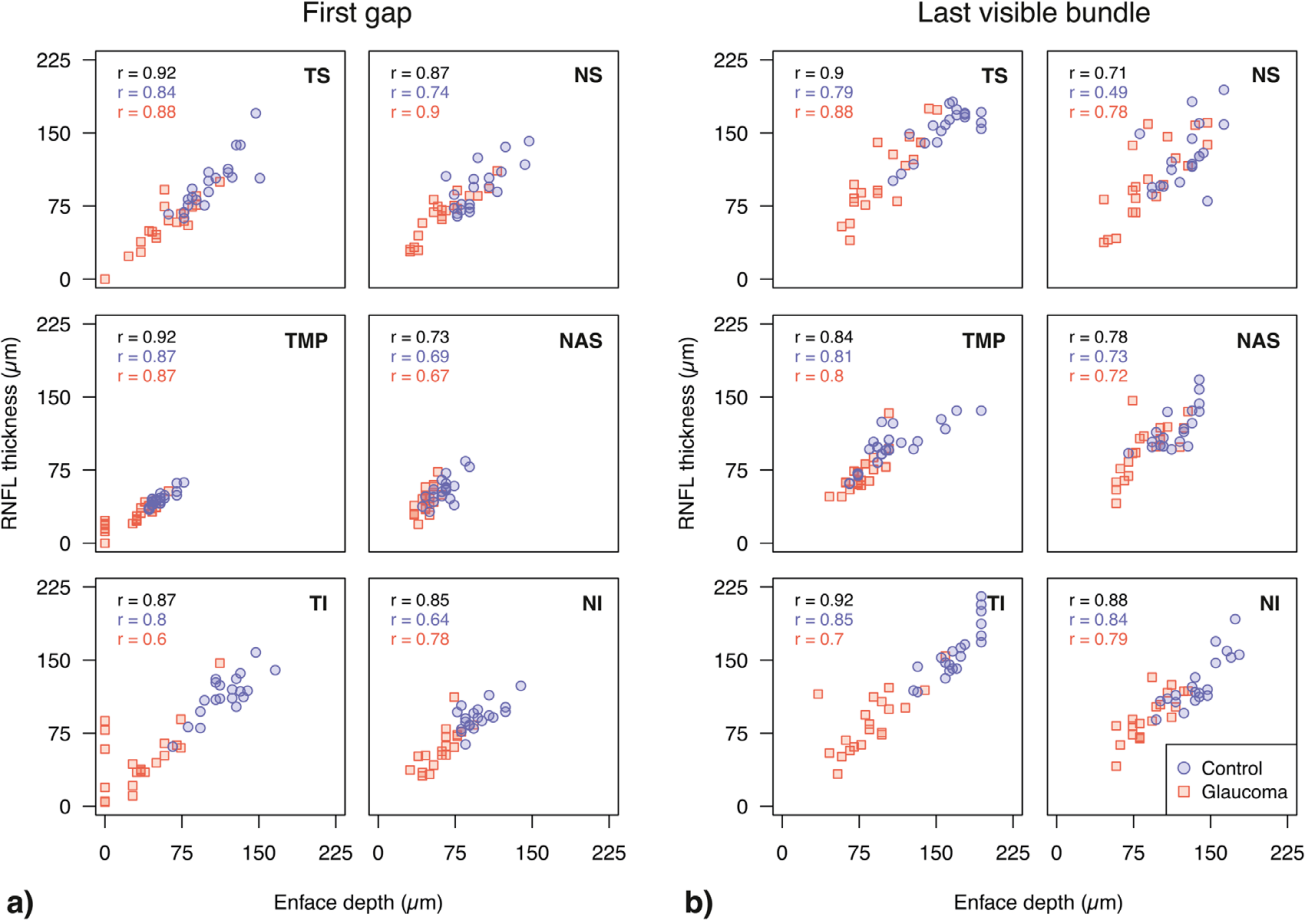
Relationships between (**a**) first gap & (**b**) last visible bundle with RNFL thickness at the corresponding angle in each sector of the ONH. Points are color coded and shaped according to disease status. The Pearson correlation coefficients are computed with data from both glaucoma and healthy eyes combined (black), as well as grouping data according to disease status (color coded accordingly). In the combined group, all correlation coefficients were *P* < 0.0001. All correlation coefficients from data grouped according to disease status were *P* < 0.001, with the exception of last visible bundle at NS and NAS in controls (*P* = 0.02 and 0.002, respectively) and NAS and NI first gap in glaucoma eyes (*P* = 0.012 and 0.005, respectively). ONH sectors are labeled as in Figure 3.

Repeated measures correlation analysis for all eyes showed a strong relationship between first gap and RNFL thickness at the same angle (*r*_df__=__199_ = 0.87, 95% CI: 0.83 to 0.90, *P* < 0.0001). Last visible bundle was also strongly correlated with corresponding RNFL thickness for all eyes (*r*_df__=__199_ = 0.78, 95% CI: 0.72 to 0.83, *P* < 0.0001). The relationship between enface indices and corresponding RNFL thickness in each ONH sector is shown in Figure 5. For first gap, the strongest correlation was found in the temporal sector (*r* = 0.92, 95% CI: 0.86 to 0.96, *P* < 0.0001), whereas the temporal inferior sector showed the strongest correlation for last visible bundle (*r* = 0.92, 95% CI: 0.85 to 0.96, *P* < 0.0001).

**Figure 6.**
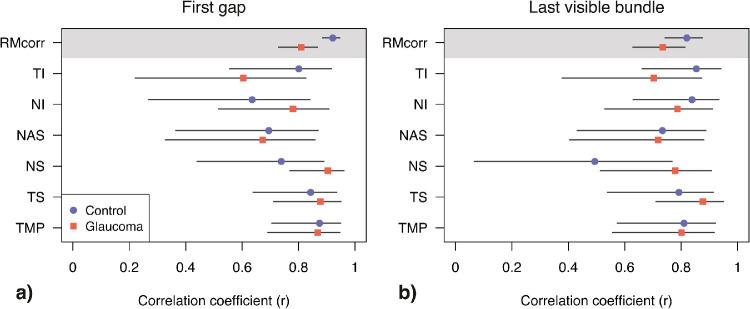
Pearson's correlation coefficients and their 95% CI for first gap (**a**) & last visible bundle (**b**) and corresponding RNFL thickness at each ONH sector. Top panel in each plot reports the overall correlation and its 95% CI limits, computed with repeated measure correlation (RMCorr). ONH sectors are labeled as in Figure 3.

To test the hypothesis that loss of reflectivity might precede thinning of the RNFL, we analyzed the strength of correlation between enface indices and RNFL thickness when grouping data according to disease status. Across all sectors, repeated measures correlation for first gap was stronger in healthy eyes compared to glaucoma eyes (*r*_df__=__99_ = 0.92, 95% CI: 0.89 to 0.95, and *r*_df__=__99_ = 0.80, 95% CI: 0.73 to 0.87, *P* < 0.0001, respectively). Overall correlation between last visible bundle and corresponding RNFL thickness was also higher in healthy eyes (*r*_df__=__99_ = 0.81, 95% CI: 0.74 to 0.88, *P* < 0.0001) than glaucoma eyes (*r*_df__=__99_ = 0.73, 95% CI: 0.62 to 0.81, *P* < 0.0001), though 95% confidence intervals overlapped. Figure 6 shows sector-wise differences in strength of correlation between glaucoma and healthy eyes. Coefficients were similar in many ONH sectors for both enface parameters, sometimes greater in healthy eyes (e.g., TI, first gap) and vice-versa in other ONH sectors (e.g., NS & NI first gap).

## Discussion

Exploiting RNFL reflectance for early glaucoma diagnosis has attracted significant research interest,^15^^,^^31^^,^^43^^–^^46^ and enface imaging now provides clinicians with a potentially powerful tool to this end.^22^^,^^24^^,^^47^^–^^49^ Enface images may facilitate a more detailed clinical approach to OCT in glaucoma than the sole consideration of thickness measurements,^13^^,^^23^^,^^27^^,^^28^ and some reports suggest that the technique might show RNFL reflectance changes before measurable thinning.^20^^,^^21^ Yet, clinical usability of enface imaging remains limited, with most objective and subjective methods for the assessment of reflectivity currently confined to research settings.^15^^,^^22^^,^^24^^,^^31^^,^^43^^,^^46^^,^^50^^,^^51^ In this study we present a simple and clinically usable method for the evaluation of glaucomatous changes in enface images, which focuses on the subjective assessment of visible presence of RNFBs around the ONH.

Enface parameters were able to identify glaucomatous changes in our sample. Both first gap and last visible bundle were significantly closer to the ILM in glaucoma, with temporal inferior sector and sectors-average measures showing greatest differences (Fig. 4). This is not surprising since the hallmark RNFL thinning in glaucoma is most easily detected in some ONH sectors including the temporal inferior sector.^11^^,^^42^^,^^52^^–^^54^ In case of thinner RNFL, deeper and hyporeflective retinal layers such as the ganglion cell layer and the inner plexiform layer would be encountered at depths closer to the ILM. Further, some preserved RNFBs in glaucoma eyes might show reduced reflectivity, hence mimicking lack of bundles and contributing to smaller enface depths in our study.^23^^,^^26^ Previous studies focused on quantitative assessment of RNFL reflectance and also found a significant effect of glaucoma, in agreement with our findings. Lower reflectivity of the RNFL was shown with time-domain OCT,^43^ and later replicated with more recent technology.^15^^,^^31^^,^^50^ Irrespective of the analysis performed, reflectivity of the RNFL was reduced in eyes with glaucoma compared to healthy eyes, and increasingly so with more severe disease.^15^^,^^31^^,^^43^^,^^50^

Diagnostic performance of enface indices was excellent in many ONH sectors (Table 2), yet statistically similar to corresponding RNFL thickness parameters. The high accuracy of conventional cpRNFL thickness suggests that glaucomatous defects in this sample were already well captured by the typical morphological OCT analysis. This is unsurprising given that our inclusion criteria required a structural defect. Nonetheless, it is notable that enface indices performed similarly to or better than conventional thickness measurements (pAUC higher in 12 of 14 comparisons, though all differences *P* > 0.05). The performance of both enface and conventional indices might not necessarily be representative of clinical settings aiming to diagnose the earliest glaucoma cases, in which conventional OCT analyses perform more poorly.^55^^–^^58^ Despite this limitation, some of the enface parameters presented hold promise for early glaucoma detection. For instance, sectors-average first gap discriminated glaucoma perfectly in this sample, warranting further exploration of the use of this parameter in glaucoma diagnosis. We also explored diagnostic accuracy in a “more difficult” subgroup of early glaucoma eyes, with MD better than −4 dB. Diagnostic performance was similar between the early subgroup and the overall sample for all indices (Supplementary Table S3), though it should be noted that these eyes still had a structural defect measurable by conventional cpRNFL thickness.

Among studies employing RNFL reflectivity for glaucoma diagnosis, none have adopted similar approaches to those proposed here. Some reports conducted quantitative analysis of RNFL reflectance for the discrimination of glaucoma with mixed results.^31^^,^^44^^,^^46^ Liu et al. compared cpRNFL thickness and a pigment epithelium normalized reflectance index of the cpRNFL for glaucoma diagnosis.^44^ Conventional thickness showed similar accuracy to reflectance indices to detect definite glaucoma, whereas some superiority of reflectivity analysis (0.05 differences in AUCs) was found for detecting glaucoma suspects.^44^ A similar normalized reflectance index was employed later by a study aiming to detect glaucoma progression.^45^ In that study, reflectance analysis did not outperform cpRNFL thickness in predicting functional progression, but, for a fixed amount of thinning, loss of reflectivity related to more rapid visual field degradation. Recently, Tan and colleagues further refined normalized reflectance indices and tested the related diagnostic capability for glaucoma compared to cpRNFL thickness.^46^ In that study, reflectance analysis outperformed thickness in terms of sensitivity at 99% specificity in both glaucoma groups. Lastly, in a case control study by Thepass and colleagues,^31^ global cpRNFL thickness outperformed measures of RNFL reflectance from the same OCT scan, both in terms of AUC (0.97 vs. 0.83) and sensitivity at 90% specificity (97% vs. 60%). Our results, therefore, align with the current evidence, which overall suggests that reflectance analysis performs well for glaucoma detection, though not likely to be substantially superior to thickness analysis. Further studies are needed to test whether reflectance information can be combined with thickness measurements to further improve OCT diagnostic accuracy. It is worth noting, though, that there remains scope for improvement in the observation and quantification of defects in enface images, and, given the strong performance of simple approaches such as ours, such improvements may yield greater diagnostic performance.

Although published data were generated with dissimilar approaches to the one used here, previous studies have shown strong correlations between RNFL thickness and reflectance.^22^^,^^31^^,^^43^^,^^46^ Our analysis also showed strong relationships between enface parameters and corresponding RNFL thickness (Fig. 5), in concordance with the literature. The reflectance-thickness relationship we found was strong but imperfect, and several reasons for incongruences should be considered. For instance, blood vessels could be expected to have a larger impact on thickness measurement, since these could be distinguished subjectively from RNFBs in enface analysis. An estimate of such effect could be inferred from the slightly poorer correlation in last visible bundle than first gap parameters (≈0.1). This may be attributable to the presence of blood vessels as major blood vessels are usually located in regions with thicker RNFL,^59^^,^^60^ where RNFBs are also expected to be visible at greater depths. First gap more often coincided with regions of thinner RNFL which are also more likely to be areas free from major blood vessels. Although methods for removal of blood vessels from OCT scans exist,^59^^,^^61^^,^^62^ they are not routinely adopted in clinics, and their usage here would likely result in further improvement of an already strong correlation. Additionally, segmentation inaccuracies of the proximal RNFL boundary could also play a role. This surface is difficult to segment,^63^ especially in areas with established damage.^15^ Since enface images only depend on the more straightforward vitreous-ILM surface segmentation these inaccuracies only affected thickness measures. The reduced dependence of enface approaches on device software's segmentation and analysis might represent additional advantages of this technique compared to thickness analysis, with the further potential of stronger interdevice comparability of results.

To explore the hypothesis that reflectivity loss precedes thinning of the RNFL, the strength of correlation was evaluated in healthy and glaucoma eyes separately. A weaker overall correlation was found in glaucoma between first gap and corresponding RNFL thickness (Fig. 6a). This was also the case for last visible bundle, though that difference was not statistically significant (Fig. 6b). A more detailed look at individual first gap sector data suggests that correlation was similar between healthy and glaucoma eyes in every sector but the temporal inferior. This was confirmed by similar repeated-measure correlation between the two groups found once censoring the temporal inferior sector from first gap data (glaucoma: *r*_df__=__79_ = 0.89, 95% CI: 0.84 to 0.93, *P* < 0.0001; and controls: *r*_df__=__79_ = 0.91, 95% CI: 0.87 to 0.94, *P* < 0.0001). The scatterplot corresponding to the aforementioned relationship (Fig. 5a, bottom left subplot) shows the presence of three outliers in an otherwise strong relationship. Enface images of these participants showed first gap in the temporal inferior sector at 0 µm below the ILM, meaning that in part of this sector there were no visible bundles, albeit still measurable RNFL thickness at corresponding angles. However, all these eyes did present significant thinning of the temporal inferior RNFL thickness, yet slightly apart from angular locations of enface first gap. It is possible that experimental settings and/or effects of blood vessels on RNFL segmentation in significantly thinned regions might have caused these observed differences. Notably, for each of these three participants, the RNFL thickness was markedly outside normal limits, allowing the device's classification system to flag this area as a defect. On the whole, data from this sample did not seem to provide compelling evidence supporting loss of reflectivity without loss of thickness of the RNFL.

Despite considerable research interest, the temporal relationship between changes of reflectivity and thickness of the RNFL is not fully understood. Findings from models of experimental glaucoma^20^^,^^21^^,^^64^^–^^66^ suggest that reflectivity deteriorates earlier than a measurable thinning of the RNFL, but evidence for a measurable time delay between reflectance and thickness changes in human glaucoma remains sparse. Overall these results fit well with findings from diagnostic accuracy studies, suggesting that analysis of RNFL reflectivity might be useful at the earliest stages of glaucoma, becoming progressively less valuable in later stages where correlation with thickness measurements is strong.^44^^,^^45^^,^^66^^,^^67^ This, however, does not preclude other uses of enface imaging providing additional value. For example, enface imaging may be useful in combination with other test modalities such as visual fields, enabling direct exploitation of the structure-function relationship without the need for spatial structure-function mapping, and in facilitating custom-perimetry based on structural data.^68^^–^^72^

Our study has limitations. We presented a novel approach for assessing glaucoma changes in enface images whose translation to practice would require little software adjunction. Clinicians could inspect the suspicious ONH sector in detail for evidence of focal loss of RNFB reflectivity and extract enface parameters. As we showed here, reduced first gap or last visible bundle seemed promising for glaucoma detection. However, additional research is needed on more diverse populations, including earliest glaucoma cases across a wider range of ages and ethnicities, to further characterize enface parameters and identify which one would be best in clinics. Further, subtle reflectivity changes may be overlooked by our subjective method, and more sensitive quantitative methods may be required to fully exploit the value of enface images.^15^^,^^22^ Second, we studied eyes with established glaucoma, and the hypothesis of discrepancies between RNFL reflectance and thickness at earlier disease stages should be considered. A more thorough longitudinal analysis on reflectivity-thickness relationship is warranted. Cross-sectional approaches such as ours adopt a reference standard for glaucoma diagnosis requiring signs (e.g., defined RNFL thinning) that bias the study in favor of tests used in the inclusion criteria. In our case, all glaucoma participants had measurable cpRNFL defects, therefore, the high performance of cpRNFL is not surprising, though this unfavorable bias does make the similar or better performance of the enface indices more noteworthy. Lastly, there are limitations due to the image processing and the grading task. We did not include correction for beam light incident angle, which is among the determinants of RNFL reflectance^73^ and is known to change in circumpapillary scans.^46^^,^^74^ We speculate that this caveat may be less detrimental to subjective evaluation of RNFB presence compared to quantification of reflectance. The enface parameters considered here could be measured with a single cube scan centered on the ONH, further reducing the impact of beam incident angle. Additionally, enface images and thickness measurements were obtained from different scans that were not mutually registered. To minimize disagreement, data in each domain were adjusted for individual fovea-disc angle, and the strong correlation found suggests that any angular incongruences were small. Concerning the grading task, the order of enface images was not randomized between glaucoma and controls, and the presence of visible glaucomatous changes in many enface images precluded masking of the grader to disease status. Further, the grading task was performed by a single observer on a single occasion. However, previous work showed both consistency between observers and repeatability within observers to be excellent in the assessment of visible presence of RNFBs.^26^^,^^48^

In conclusion, our simple method to observe visible presence of RNFBs reliably identified glaucomatous defects in enface OCT images, with diagnostic performance at least as good as existing thickness parameters. No strong evidence of reflectivity loss without corresponding thickness loss was found. Development of more sensitive automated analyses and integration with perimetry may realize further potential of enface OCT images in glaucoma.

## Supplementary Material

Supplement 1

Supplement 2
