# Supplementary material for: A Simple Subjective Evaluation of Enface OCT Reflectance Images Distinguishes Glaucoma From Healthy Eyes
Source: Transl Vis Sci Technol. 2021 May 25;10(6):31. doi: 10.1167/tvst.10.6.31 (PMC8161697; doi:10.1167/tvst.10.6.31)
Supplement: Supplement 2 [file tvst-10-6-31_s002.pdf]

Supplementary material

S1 – Animated version of Figure 1, showing how visible presence of RNFBs change at different depths in healthy and glaucoma eyes

S2 – Boxplots showing pairwise differences between glaucoma and control eyes for RNFL thickness at corresponding angles of enface first gap (a) and last visible bundle (b). As per Figure 4, differences were considered significant when p<0.0036 (after Bonferroni correction, 14 comparisons), and flagged with (*). Data are reported for every ONH sector and the sectors-average. ONH sectors acronyms as per Figure 4.

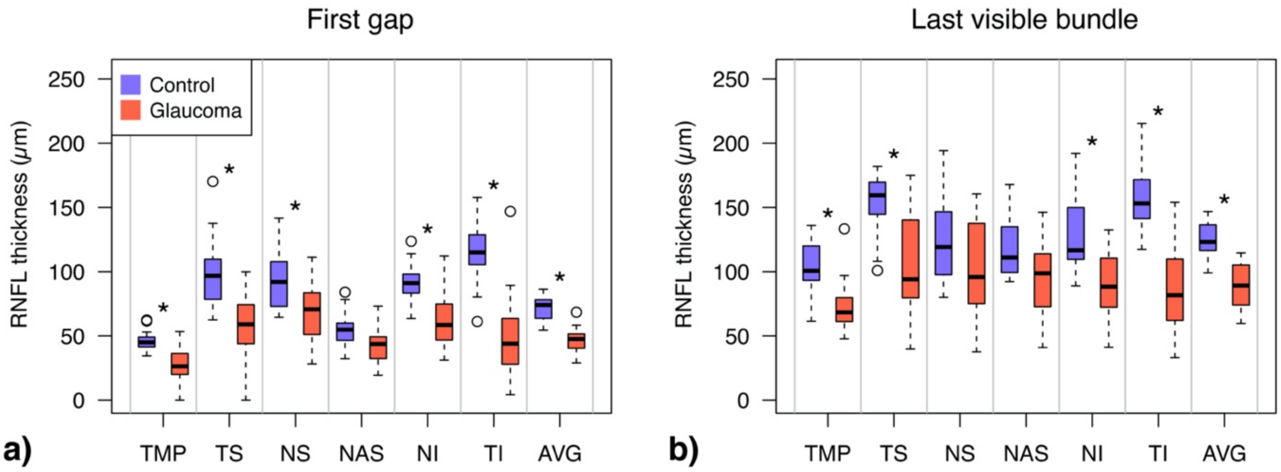

S3 - Supplementary table

Table S3. Diagnostic accuracy analysis (standardised pAUC at 90-100% specificity with 95% CIs) repeated in a subgroup of early glaucoma participants with MD better than -4.0dB (n=11). ONH sector labels as per Figure 3.

**ONH sector**	**Enface First gap**	**RNFLT at First Gap Angle**	**Enface Last Visible**	**RNFLT at Last Visible Angle**	**cpRNFL thickness**
TMP	0.83 (0.68, 0.95)	0.81 (0.67, 0.98)	0.55 (0.47, 0.88)	0.67 (0.52, 0.93)	0.67 (0.53, 0.96)
TS	0.70 (0.55, 0.89)	0.71 (0.57, 0.90)	0.73 (0.57, 0.92)	0.67 (0.52, 0.86)	0.62 (0.52, 0.86)
NS	0.76 (0.62, 0.90)	0.62 (0.47, 0.79)	0.69 (0.55, 0.86)	0.61 (0.52, 0.79)	0.65 (0.52, 0.81)
NAS	0.65 (0.52, 0.94)	0.64 (0.52, 0.83)	0.65 (0.51, 0.86)	0.64 (0.52, 0.83)	0.65 (0.52, 0.86)
NI	0.84 (0.69, 0.98)	0.76 (0.57, 1)	0.71 (0.57, 0.90)	0.69 (0.57, 0.90)	0.90 (0.76, 1)
TI	0.98 (0.90, 1)	0.90 (0.76, 1)	1.00 (1, 1)	0.95 (0.86, 1)	0.98 (0.90, 1)
AVG	1.00 (1, 1)	0.98 (0.9, 1)	0.86 (0.66, 1)	0.76 (0.62, 0.95)	0.95 (0.86, 1)
